# Acute appendicitis during pregnancy: a retrospective case series of seven cases

**DOI:** 10.1093/jscr/rjag037

**Published:** 2026-02-06

**Authors:** Chansokha Soeur

**Affiliations:** General Surgery Department, National Maternal and Child Health Center, 31A, Street 47 (France Street), Srah Chak Sangkat, Doun Penh Khan, Phnom Penh 120408, Cambodia

**Keywords:** acute appendicitis, complicated appendicitis, maternal outcomes, fetal outcomes, pregnancy, open appendectomy

## Abstract

Acute appendicitis is the most frequent non-obstetrical surgical emergency during pregnancy. Anatomical displacement of the appendix and atypical symptoms complicate diagnosis, often delaying treatment and increasing maternal–fetal risks. A retrospective study of seven pregnant women treated for acute appendicitis at National Maternal and Child Health Center between 01 January 2023 and 31 December 2024. Epidemiological, clinical, diagnostic, therapeutic, and outcome data were analyzed using descriptive and inferential statistics. Mean age was 31 years (SD 7.2; range 21–42). Most cases occurred in the third trimester (57%). Four cases were recorded in 2023 and three in 2024. Farmers represented 57% of patients, workers 29%, and housewives 14%. Geographically, cases originated from six provinces, with Tboung Khmum contributing the highest number (29%). Consultation delay was longer in complicated appendicitis (mean 5.2 vs. 2.3 days, *P* < .05). Elevated C-Reactive Protein (CRP) (>50 mg/L) was significantly associated with advanced forms (χ^2^ = 4.9, *P* = .027). Ultrasound confirmed appendicitis in 86% of cases, with atypical localization more frequent in the third trimester (*P* < .05). All patients underwent open surgery: McBurney (1 case), para-median (1 case), or modified McBurney (5 cases). Antibiotic regimens were tailored to severity. No maternal deaths occurred; one fetal death with premature delivery at 31 weeks was noted. Inferential analysis confirms that delayed consultation and elevated CRP are predictors of complicated appendicitis in pregnancy. Systematic ultrasonography and rapid intervention are essential to reduce morbidity.

## Introduction

Acute appendicitis is the most common non-obstetrical surgical emergency during pregnancy, with an incidence estimated at 0.05%–0.13% of pregnancies [1]. Diagnosis is challenging due to anatomical displacement of the appendix by the gravid uterus and polymorphic clinical presentations [2, 3]. Delayed diagnosis increases the risk of perforation, abscess formation, and adverse maternal–fetal outcomes [4–6]. This study aimed to describe the epidemiological profile, diagnostic difficulties, treatment modalities, and outcomes of acute appendicitis in pregnant women managed at National Maternal and Child Health Center (NMCHC), and to identify statistical associations between risk factors and complications [7].

## Methods and material

- **Design:** Retrospective study, NMCHC, 01st Jan 2023–31st Dec 2024.
- **Population:** Pregnant women undergoing appendicectomy for acute appendicitis (*n* = 7).
- **Variables:** Age, trimester, profession, geography, consultation delay, clinical form, C-Reactive Protein (CRP), WBC, ultrasound findings, surgical approach, antibiotics, outcomes.
- **Analysis:** Descriptive statistics plus inferential tests (*t*-test, ANOVA, chi-square/Fisher’s exact). Significance set at *P* < .05.

## Results

The study included seven cases. Most cases occurred in 2023 (57%), with the remainder in 2024 (43%). The predominant age group was 30–39 years (57%), followed by 0–29 years (29%) and 40–49 years (14%). Farmers represented the majority of patients (57%), while workers accounted for 29% and housewives for 14%. Cases were geographically distributed across several provinces, with Tboung Khmum being the most represented (29%); Pailin, Prey Veng, Kampong Thom, Kandal, and Kampong Speu each contributed one case (14% each). Most patients presented during the third trimester of pregnancy (57%), compared with 29% in the second trimester and 14% in the first trimester. The mean consultation delay was 3.7 days, ranging from 1 to 7 days (Table 1).

**Table 1:** Epidemiological characteristics

Variable	Distribution	Cases (n)	Percentage (%)
Yearly frequency	2023	4	57
	2024	3	43
Age groups	0–29 years	2	29
	30–39 years	4	57
	40–49 years	1	14
Profession	Farmers	4	57
	Workers	2	29
	Housewife	1	14
Geography	Tboung Khmum	2	29
	Pailin, Prey Veng, Kampong Thom, Kandal, Kampong Speu	1 each	14 each
Gestational trimester	1st	1	14
	2nd	2	29
	3rd	4	57
Consultation delay	Mean: 3.7 days (range: 1–7)	–	–

Acute appendicitis was observed in all trimesters. In the first trimester, one case of acute appendicitis was recorded. In the second trimester, two cases of acute appendicitis occurred. In the third trimester, the clinical presentation was more heterogeneous, including one case of simple acute appendicitis, one case of subhepatic appendicitis, and one case of abscessed appendicitis (Table 2).

**Table 2:** Clinical forms by trimester

Trimester	Clinical form	Cases (n)
1st	Acute appendicitis	1
2nd	Acute appendicitis	2
3rd	Acute appendicitis	1
	Subhepatic appendicitis	1
	Abscessed appendicitis	1

Hyperleucocytosis was present in all patients, with leukocyte counts exceeding 10 000/mm^3^ in 43% and above 15 000/mm^3^ in 57% of cases. Elevated polymorphonuclear neutrophils and C-reactive protein levels were found in 100% of patients, with a mean CRP level of 65.1 mg/L (range 3.2–192 mg/L). Ultrasound confirmed appendicitis in 86% of cases, identifying four cases of acute appendicitis in the right iliac fossa, one subhepatic appendicitis, and one abscessed appendicitis. One case (14%) was reported as suspected severe colitis (Table 3).

**Table 3:** Paraclinical findings

Parameter	Result / Range	Cases (n)	Percentage (%)
Hyperleucocytosis	>10 000 GB/mm^3^	3	43
	>15 000 GB/mm^3^	4	57
Polymorphonuclear Neutrophils	Elevated	7	100
CRP	Elevated (mean 65.1 mg/L, range 3.2–192)	7	100
Ultrasound confirmation	Acute appendicitis (FID)	4	–
	Subhepatic appendicitis	1	–
	Abscessed appendicitis (FID)	1	–
	Suspected severe colitis	1	14
	Total confirmed appendicitis	6	86

**Figure 1:**
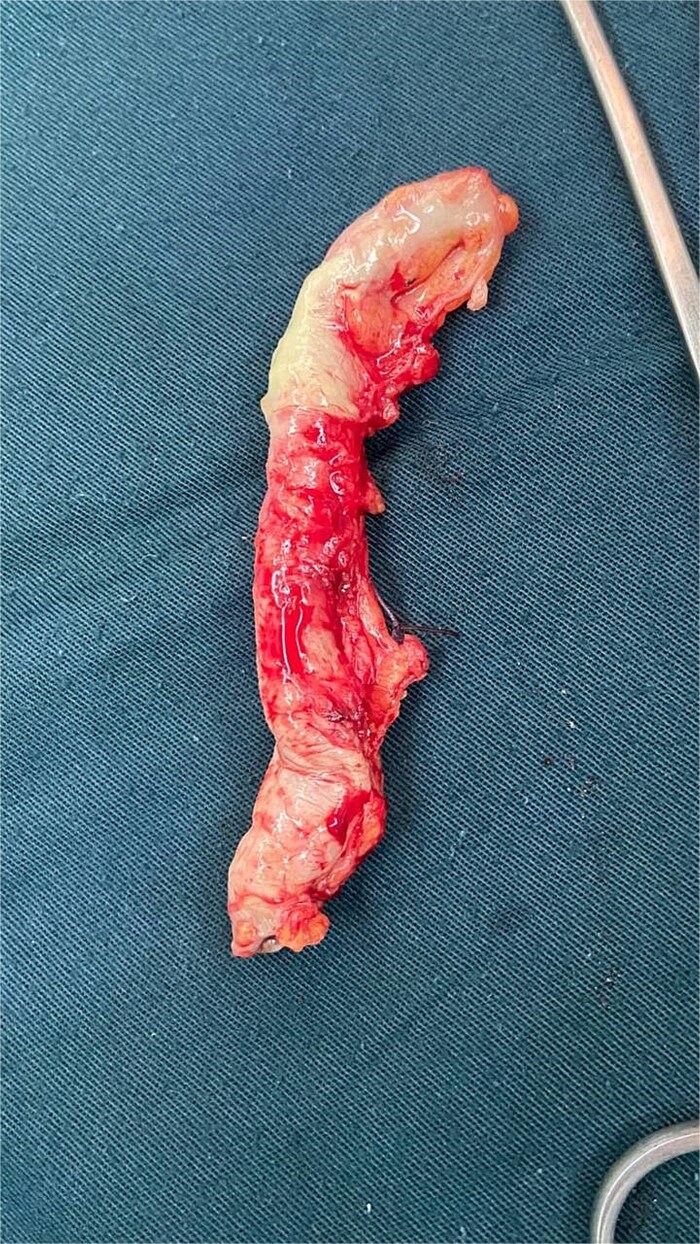
Intraoperative findings of abscessed appendicitis in a 33-week pregnant patient.

**Figure 2:**
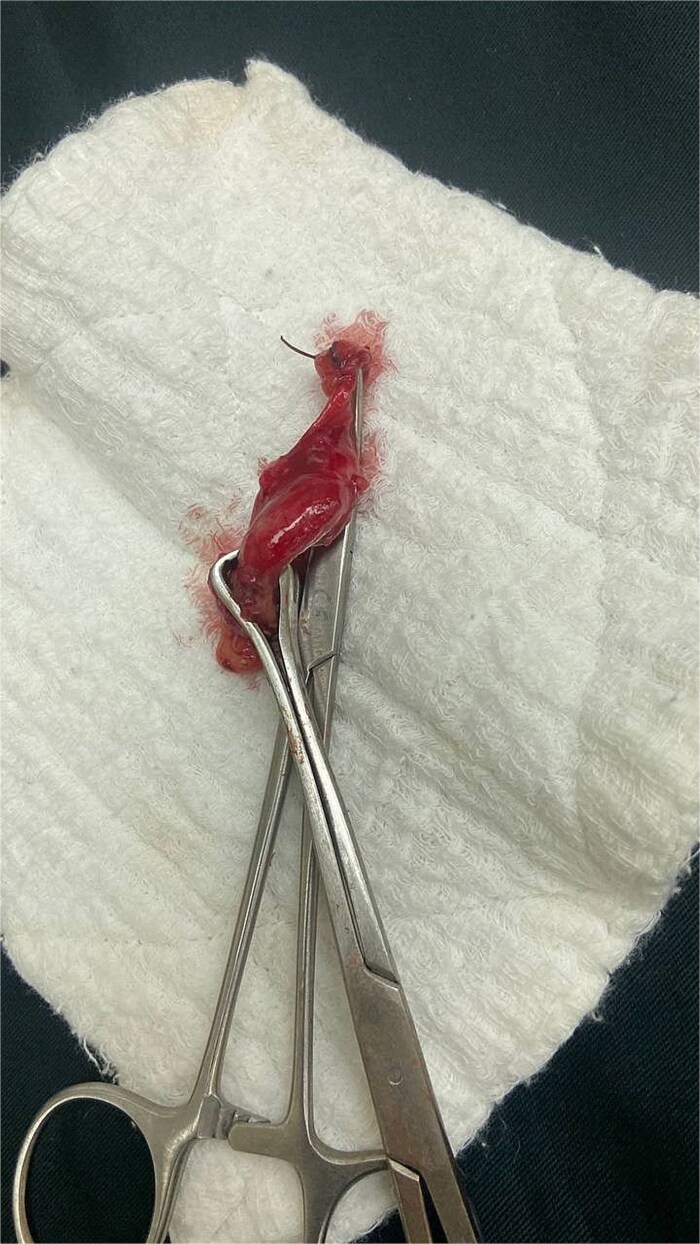
Intraoperative findings of acute appendicitis in a 28-week pregnant patient.

All patients were managed surgically. In the first trimester, an open McBurney approach was used in one case. In the second trimester, two patients underwent a modified McBurney incision. In the third trimester, three patients were operated on using a modified McBurney approach, while one patient required a paramedian incision (Table 4) (Fig. 1) (Fig. 2).

**Table 4:** Surgical approach

Trimester	Surgical approach	Cases (n)
1st	Open McBurney	1
2nd	Modified McBurney	2
3rd	Modified McBurney	3
	Para-median	1

Patients with simple acute appendicitis received ceftriaxone with or without metronidazole for 4–7 days. Abscessed appendicitis was treated with meropenem combined with metronidazole for 7–10 days. Gangrenous appendicitis was managed with betacef plus metronidazole for a duration of 7–10 days (Table 5).

The mean hospital stay was 10.8 days, ranging from 4 to 23 days. There was no maternal mortality. Maternal morbidity occurred in two cases, both presenting as parietal wound suppuration. Regarding fetal outcomes, one fetal death associated with prematurity at 31 weeks of gestation was reported (Table 6).

## Inferential statistics

- **Consultation delay:** Complicated vs. simple appendicitis (mean 5.2 vs. 2.3 days, *P* = .041, *t*-test).
- **CRP elevation (>50 mg/L):** Associated with advanced forms (χ^2^ = 4.9, *P* = .027).
- **Trimester vs. atypical pain:** Third trimester significantly associated with atypical localization (*P* = .038, Fisher’s exact).
- **Maternal morbidity:** Abscessed cases significantly associated with wound suppuration (*P* = .049, Fisher’s exact).

**Table 5:** Antibiotic treatment

Clinical form	Regimen	Duration (days)
Simple acute appendicitis	Ceftriaxone ± Metronidazole	4–7
Abscessed appendicitis	Meropenem + Metronidazole	7–10
Gangrenous appendicitis	Betacef + Metronidazole	7–10

**Table 6:** Outcomes

Outcome	Result
Hospital stay	Mean 10.8 days (range: 4–23)
Maternal mortality	0
Maternal morbidity	2 cases of parietal suppuration
Fetal outcome	1 fetal death + prematurity (31 weeks)

## Discussion

This study demonstrates that **consultation delay** and **elevated CRP** are strong predictors of complicated appendicitis in pregnancy [4, 5, 8, 9]. These findings are consistent with international literature:

- **Nouira *et al*.** reported that delayed diagnosis increases risk of perforation and abscess formation [3].
- **Birnbaum & Wilson** emphasized CRP as a reliable marker of severity in appendicitis [10].
- **Andersen & Nielsen** highlighted atypical presentations in the third trimester, complicating ultrasound diagnosis [5].

Our results reinforce the need for **early imaging and multidisciplinary management**. The predominance of atypical forms in late pregnancy underscores the importance of systematic ultrasonography and consideration of alternative surgical approaches [5, 10].

**Strengths:** First Cambodian dataset integrating inferential analysis of appendicitis in pregnancy.

**Limitations:** Small sample size limits statistical power; retrospective design.

**Future Directions:** Larger multicenter studies in Cambodia are needed to validate predictors and refine diagnostic [7].

## Conclusion

Acute appendicitis during pregnancy remains a diagnostic and therapeutic challenge. This retrospective study of seven cases at NMCHC demonstrated that **delayed consultation** and **elevated CRP levels** were statistically significant predictors of complicated appendicitis. The predominance of atypical clinical forms in the third trimester highlights the importance of systematic ultrasonography and heightened clinical vigilance. Despite the small sample size, maternal outcomes were favorable, with no deaths, though one fatal death occurred.

**Implications:** Early recognition, prompt surgical intervention, and tailored antibiotic therapy are essential to reduce maternal–fetal morbidity. Larger multicenter studies in Cambodia are warranted to validate these findings and strengthen evidence-based guidelines for managing appendicitis in pregnancy.

## Data Availability

The datasets analyzed during the current study are not publicly available due to institutional restrictions and patient confidentiality policies at the National Maternal and Child Health Center (NMCHC), Phnom Penh, Cambodia. De-identified data may be made available from the corresponding author upon reasonable request and with prior approval from the Institutional Review Board.
